# Neutralizing antibody activity in convalescent sera from infection in humans with SARS-CoV-2 and variants of concern

**DOI:** 10.1038/s41564-021-00974-0

**Published:** 2021-10-15

**Authors:** Liane Dupont, Luke B. Snell, Carl Graham, Jeffrey Seow, Blair Merrick, Thomas Lechmere, Thomas J. A. Maguire, Sadie R. Hallett, Suzanne Pickering, Themoula Charalampous, Adela Alcolea-Medina, Isabella Huettner, Jose M. Jimenez-Guardeño, Sam Acors, Nathalia Almeida, Daniel Cox, Ruth E. Dickenson, Rui Pedro Galao, Neophytos Kouphou, Marie Jose Lista, Ana Maria Ortega-Prieto, Harry Wilson, Helena Winstone, Cassandra Fairhead, Jia Zhe Su, Gaia Nebbia, Rahul Batra, Stuart Neil, Manu Shankar-Hari, Jonathan D. Edgeworth, Michael H. Malim, Katie J. Doores

**Affiliations:** 1 Department of Infectious Diseases, School of Immunology and Microbial Sciences, King’s College London, London, UK; 2 Centre for Clinical Infection and Diagnostics Research, Department of Infectious Diseases, Guy’s and St Thomas’ NHS Foundation Trust, London, UK

**Keywords:** Viral infection, SARS-CoV-2

## Abstract

COVID-19 vaccine design and vaccination rollout need to take into account a detailed understanding of antibody durability and cross-neutralizing potential against SARS-CoV-2 and emerging variants of concern (VOCs). Analyses of convalescent sera provide unique insights into antibody longevity and cross-neutralizing activity induced by variant spike proteins, which are putative vaccine candidates. Using sera from 38 individuals infected in wave 1, we show that cross-neutralizing activity can be detected up to 305 days pos onset of symptoms, although sera were less potent against B.1.1.7 (Alpha) and B1.351 (Beta). Over time, despite a reduction in overall neutralization activity, differences in sera neutralization potency against SARS-CoV-2 and the Alpha and Beta variants decreased, which suggests that continued antibody maturation improves tolerance to spike mutations. We also compared the cross-neutralizing activity of wave 1 sera with sera from individuals infected with the Alpha, the Beta or the B.1.617.2 (Delta) variants up to 79 days post onset of symptoms. While these sera neutralize the infecting VOC and parental virus to similar levels, cross-neutralization of different SARS-CoV-2 VOC lineages is reduced. These findings will inform the optimization of vaccines to protect against SARS-CoV-2 variants.

## Abstract (web summary)

The authors assess the durability and long-term cross-reactivity of neutralizing antibodies raised in response to infections with SARS-CoV-2 or variants of concern in humans.

## Main

Neutralizing antibodies against the spike glycoprotein of severe acute respiratory syndrome coronavirus 2 (SARS-CoV-2) are important in protection against re-infection and/or severe disease^1–6^. An important component of vaccines that protect against COVID-19 is the elicitation of neutralizing antibodies that bind the SARS-CoV-2 spike protein. A major challenge in controlling the COVID-19 pandemic will be the elicitation of a durable neutralizing antibody response that also provides protection against emerging variants of SARS-CoV-2. While the kinetics and correlates of the neutralizing antibody response have been extensively studied in the early phase following SARS-CoV-2 infection^7–12^, information on the durability and long-term cross-reactivity of the antibody response against SARS-CoV-2 following infection and/or vaccination is limited due to its recent emergence in the human population and large-scale COVID-19 vaccination only being initiated in December 2020.

We have previously studied the antibody response in SARS-CoV-2-infected healthcare workers and in hospitalized individuals in the first 3 months following infection using longitudinal samples^8^. We showed that the humoral immune response was typical of that following an acute viral infection whereby the sera neutralizing activity peaked around 3–5 weeks post onset of symptoms (POS) and then declined as the short-lived antibody-secreting cells die^3^. However, it remained to be seen whether the neutralizing antibody response would continue to decline after the first 3 months POS or reach a steady state. In the absence of current long-term COVID-19 vaccine follow-up, knowledge of the longevity of the neutralizing antibody response acquired through natural infection with ancestral SARS-CoV-2 during wave 1 of the COVID-19 pandemic at late time points (up to 10 months POS) may provide important indicators for the durability of vaccine-induced humoral immunity.

SARS-CoV-2 variants encoding mutations in the spike protein have been identified and include B.1.1.7 (Alpha variant, initially reported in the United Kingdom)^13^, P.1 (Gamma variant, first reported in Brazil), B.1.351 (Beta variant, first reported in South Africa)^14^ and B.1.617.2 (Delta variant, first reported in India)^15^, which have been associated with more efficient transmission^16–18^. Mutations of particular concern for vaccine immunity are those present in the receptor binding domain (RBD) of the spike protein, which is a dominant target for the neutralizing antibody response^19–22^. Despite B.1.1.7, P.1, B.1.351 and B.1.617.2 showing increased resistance to neutralization by convalescent and vaccinee sera collected at the peak of the antibody response^20,23–39^, cross-neutralizing activity has been observed. In contrast, complete loss of neutralization has been observed for some monoclonal antibodies (mAbs) targeting specific epitopes on either the amino-terminal domain (NTD) or the RBD of the spike protein^20,25,27,28,40^. Combined, these studies indicate that mutations in the spike protein may be arising in part due to the selective pressure of neutralizing antibodies in convalescent plasma^41–43^. To counter such mutations and their attendant antigenic changes, vaccines using the spike proteins from these variants of concern (VOCs) are under investigation^44–47^. Whether the variant spike proteins will elicit a robust neutralizing response with superior cross-neutralizing activity against parental strains and newly emerging variants has not been extensively studied^29,48,49^. Natural infection provides an important opportunity to compare the neutralizing antibody titres and cross-neutralizing activity generated from individuals exposed to different spike variants and will give insights into the antigenic distance between spike variants, thereby informing the design of second-generation vaccine candidates based on VOCs.

We set out to investigate the longevity of the neutralizing and cross-neutralizing antibody response against viral variants from wave 1 infections up to 10 months POS, the immunogenicity of the B.1.1.7, B.1.351 and B.1.617.2 spike variants in natural infection, and the antigenic distance between SARS-CoV-2 VOCs. We collected sera in an observational study between 145 and 305 days POS from individuals infected in wave 1 who were hospitalized patient and healthcare worker cohorts^8^, as well as sera from individuals with a confirmed B.1.1.7, B.1.351 or B.1.617.2 infection up to 73 days POS. We analysed the neutralizing potential of these sera against SARS-CoV-2 and a range of VOCs.

## Results

### Persistence of spike IgG POS

**Fig. 1:**
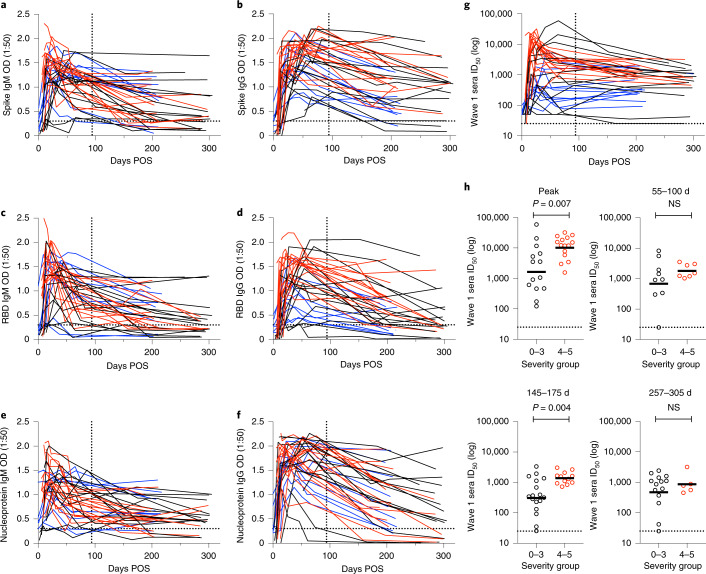
Serum spike protein IgG binding and neutralizing activity is sustained up to 305 days POS. **a**–**f**, ELISAs were used to assess the binding of IgM to the spike protein (**a**), IgG to the spike protein (**b**), IgM to the RBD (**c**), IgG to the RBD (**d**), IgM to the nucleoprotein (**e**) and IgG to the nucleoprotein (**f**). Recombinant spike protein and the RBD are the Wuhan-1 strain. Sera were diluted to 1:50 and samples were run in duplicate and background subtracted. Each line represents one individual (*n* = 39), and they are colour coded as follows: red, disease severity 4–5; black, disease severity 0–3; blue, healthcare workers. The vertical dotted line indicates the end of the time period that was studied in our original analysis of this cohort^8^. The horizontal dotted line represents the ELISA cut-off for seropositivity. **g**, Neutralization (ID_50_) measured against HIV-1 pseudotyped virus particles expressing the Wuhan-1 spike protein (WT). The vertical dotted line indicates the latest time point studied in our original analysis of this cohort^8^. The horizontal dotted line represents the cut-off for the assay. **h**, Comparison of the geometric mean ID_50_ between individuals experiencing 0–3 (black) or 4–5 (red) disease severity for the highest neutralization titre measured (peak, *n* = 29) and at different later time points POS (55–100 days*, n* = 16 sera; 145–175 days, *n* = 29 sera; 257–305 days, *n* = 19 sera). *P* values were calculated using Mann–Whitney two-sided *U*-test. NS, not significant. The line represents the geometric mean ID_50_ for each group. Source data

We previously reported^8^ antibody responses in sera up to 3 months POS in hospitalized patients and healthcare workers experiencing a range of COVID-19 severity, from asymptomatic infection to requiring extracorporeal membrane oxygenation. Additional serum samples were collected at time points >100 days POS from any individuals who returned to hospital as part of their routine clinical care (a subset comprising 29 out of 59 participants), in addition to healthcare workers still employed at St Thomas’ Hospital (a subset comprising 9 out of 37 participants). No participants had received the COVID-19 vaccine at the time of serum collection. In total, 64 serum samples were collected from 38 individuals, including 16 sera collected between 145 and 175 days POS (TP3), 29 collected between 180 and 217 days (TP4) and 19 collected between 257 and 305 days POS (TP5). We first determined the presence of IgM and IgG against the spike protein, the RBD and the nucleoprotein in patient sera collected at >100 days POS (Fig. 1a–f). Optical density (OD) values were measured for sera diluted at 1:50. Although the IgM response decreased to low levels against the spike protein, the RBD, and the nucleoprotein at later time points, IgM was still detected against all three antigens in some individuals. The IgG response also decreased over time to some extent for most individuals, but remained detectable at time points up to ~300 days POS. Those with IgG OD values near to baseline spanned across all disease severity groups.

We previously used pre-COVID-19 control sera to set a threshold OD value of fourfold above background as a cut-off for SARS-CoV-2 seropositivity^50^. Using this cut-off, 5 out of 45 (11.1%) and 3 out of 19 (15.8%) of individuals had IgG levels below the cut-off against all three antigens (the spike protein, the RBD and the nucleoprotein) between 145 and 217 days POS (TP3 and TP4) and 257 and 305 days POS (TP5), respectively. The lowest seroreactivity was observed against RBD at time points >145 days POS. An IgG response to the nucleoprotein has been used as an indicator of previous SARS-CoV-2 infection when studying COVID-19 vaccine responses^51,52^. However, at >145 days POS, 17 out of 64 (26.6%) of sera had an OD value against the nucleoprotein that was below this threshold. This suggests that a complementary or alternative SARS-CoV-2 antigen is needed to improve the determination of previous virus exposure in the context of vaccination for individuals infected >6 months previously.

### Neutralizing antibody responses in convalescent sera

**Extended Data Fig. 1:**
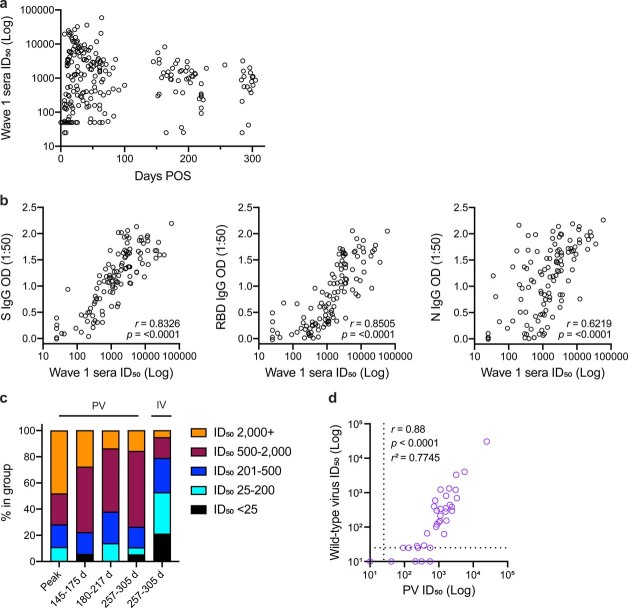
Neutralizing antibodies persist for up to 10 months post onset of symptoms. **a**) ID_50_ of neutralization for all wave 1 sera included in Fig. 1g. **b**) Correlation between ID_50_ (measured against spike pseudotyped virus) and either optimal density of IgG binding to S, RBD or N. (*r*^*2*^ = 0.6942), RBD (*r*^*2*^ = 0.6250) and N protein (*r*^*2*^ = 0.3861) (Spearman’s correlation, two-tailed, *r*; a linear regression was used to calculate the goodness of fit, *r*^*2*^). **c**) Percentage of individuals in each time window with undetectable (ID_50_ < 25), low (ID_50_ 25 – 200), medium (ID_50_ 201 – 500), high (ID_50_ 501 – 2,000) or potent (ID_50_ 2,000 + ) neutralizing antibody titres. The peak neutralization time point (n = 110) includes hospitalized patients and healthcare workers reported in Seow *et al*^8^, as well as 14 additional donors reported in this study. The time point from the longitudinal samples with the peak ID_50_ was used in ‘peak’. The first four bars show ID_50_ values measured against wild-type Spike pseudotyped virus (PV). The final bar show ID_50_ values measured against infectious SARS-CoV-2 virus (IV) for sera collected between 257-305 days POS. **d)** Correlation between ID_50_ values measured using wild-type infectious virus and pseudotyped virus for selected sera (n = 36) (Spearman correlation, two-tailed, r). A linear regression was used to calculate the goodness of fit (r^2^). The dotted lines represent the lowest serum dilution used in each assay. Sera which did not reach 50% neutralization at 1:25 dilution are given a value of 10 and is not included in the correlation. Source data

The longevity of the neutralizing activity in patient sera was measured using HIV-1-based virus particles, pseudotyped with the SARS-CoV-2 Wuhan-1 spike protein (referred to as wild type (WT)) (Fig. 1g and Extended Data Fig. 1a). Our previous study^8^ had shown a decline in neutralizing antibody titre (ID_50_, the serum dilution that inhibits 50% infection) in the first 3 months following SARS-CoV-2 infection, but whether the titre would reach a steady level was not determined. The neutralization potency of matched longitudinal sera collected at time points up to 305 days POS revealed that the rate of decline in neutralization activity slowed in the subsequent 4–7-month period, and neutralizing activity could readily be detected in 18 out of 19 of the sera tested between 257 and 305 days POS, with a geometric mean titre (GMT) of 640. Enzyme linked immunosorbent assay (ELISA) OD values for spike IgG, RBD IgG and nucleoprotein IgG correlated well with the ID_50_ (of neutralization) (Extended Data Fig. 1b). A cross-sectional analysis of all the wave 1 sera showed that the GMT at 145–175, 180–217 and 257–305 days POS decreased from 1,199 to 635 and 640, respectively. The percentage of donors displaying potent neutralization (ID_50_ > 2,000) was 48.2% at peak neutralization (as previously determined in Seow et al.^8^) and this decreased to 27.8%, 13.8% and 15.8% at 145–175, 180–217 and 257–305 days POS, respectively (Extended Data Fig. 1c). Neutralization of selected sera (*n* = 36) was also tested against live virus (strain England 02/2020/407073) using Vero-E6 TMPRSS2 cells^53^ as the target cell line. As previously observed, ID_50_ values against live virus correlated well with the ID_50_ values against spike pseudotyped particles^8,20^ (Extended Data Fig. 1d). Neutralization was detected in 15 out of 19 samples tested between 257 and 305 days POS (Extended Data Fig. 1c).

We had previously observed that individuals experiencing the most severe disease had higher peak neutralization titres^8^. Consistent with this, we observed higher mean peak ID_50_ values for those with most severe disease, as well as higher GMTs at 145–175, 180–217 and 257–305 days POS, although this trend was not always statistically significant (Fig. 1h). A wider heterogeneity in the magnitude of the neutralizing antibody response in the 0–3 severity group was seen at all time points studied compared with the 4–5 severity group.

Overall, the neutralizing antibody response following SARS-CoV-2 infection can persist for up to 10 months POS.

### Cross-neutralizing activity against SARS-CoV-2 VOCs

**Fig. 2:**
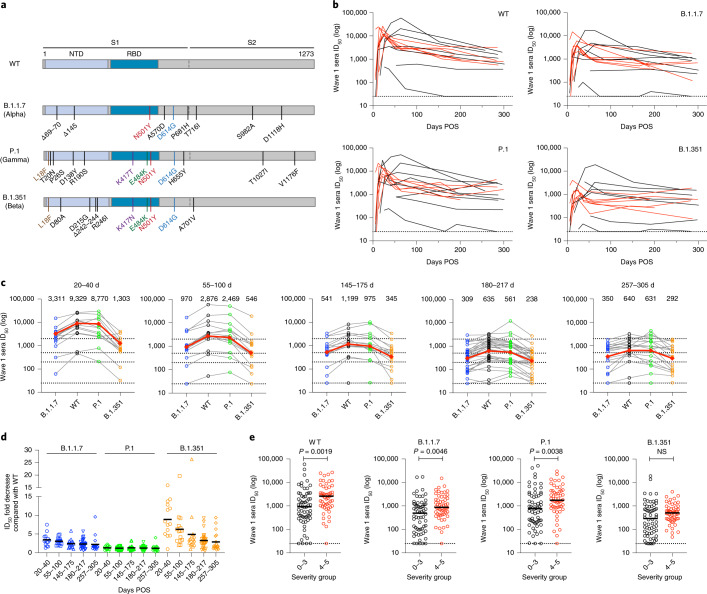
Sera from wave 1 shows cross-neutralization of SARS-CoV-2 VOCs. **a**, Schematic showing the position of spike mutations in the B.1.1.7 (Alpha), P.1 (Gamma) and B.1.351 (Beta) variants. The major spike domains are indicated. **b**, Neutralization by longitudinal wave 1 sera against WT, B.1.1.7, P.1 and B.1.351 spike pseudotyped virus. Neutralization is shown for 14 individuals, colour coded as follows: red, disease severity 4–5; black, disease severity 0–3. **c**, Neutralization of sera, collected within five different time periods, against the four SARS-CoV-2 spike variants. GMTs against each virus are shown on the top of each panel. Each dot represents one individual, and matched data points for neutralization of different spike variants by the same serum sample are connected by lines. A maximum of one sample for each individual is included within each time period. Neutralization of B.1.1.7 is shown in blue, WT in black, P.1 in green and B.1.351 in orange. The dotted lines represent the neutralization cut-offs used to determine no, low, medium, high and potent neutralization (Extended Data Fig. 1c). The red line represents the GMT against the four viruses (GMT values listed above the graph columns). **d**, Fold reductions in neutralization as compared with the WT spike pseudotyped virus at the five time points (*n* = 74 sera in total). The horizontal black lines represent the average fold reduction. **e**, Comparison of the GMT between those with 0–3 (black) and 4–5 (red) disease severity for the four variants. All sera collected up to 305 days POS are included in this analysis (*n* = 116). *P* values were calculated using Mann–Whitney two-sided *U*-test. The horizontal lines represent the geometric mean ID_50_ for each group. Source data

Initially, longitudinal sera collected from 14 individuals between days 6 and 305 POS were used to compare the magnitude and kinetics of neutralizing activity against WT SARS-CoV-2, B.1.1.7, P.1 and B.1.351 variant spike pseudotyped particles (Fig. 2a). The kinetics of neutralizing activity in sera were similar against all four variants, and a peak in neutralization was observed around 3–5 weeks POS followed by decline to a steady level of neutralization (Fig. 2b).

Having observed similar kinetics in the neutralization of VOCs, we focused on the extent of cross-neutralizing activity of wave 1 sera collected at later time points (145–305 days POS). Neutralization titres (ID_50_) against the four variants were measured (*n* = 66) and the fold-change in ID_50_ compared with the WT for each variant was compared within five time windows: acute (20–40 days POS), 55–100, 145–175, 180–217 and 257–305 days POS (Fig. 2c). Neutralization potency against the P.1 variant was most similar to neutralization potency against the WT virus at all five time points, with an average reduction in ID_50_ ranging from 1.2-fold to 1.3-fold (Fig. 2d). In contrast, and similar to previous reports^20,23–30^, both B.1.1.7 and B.1.351 were more resistant to neutralization at all time points, with the greatest decrease in neutralization observed for B.1.351. At later time points, the mean fold-change in neutralization ID_50_ for both the B.1.1.7 and B.1.351 variants compared with the WT ID_50_ was decreased in magnitude (Fig. 2d), which suggests that continued antibody maturation and improved tolerance to spike mutations are occurring. For example, the average fold reduction in ID_50_ against B.1.351 was 8.9-fold in the acute phase, and this decreased to 2.9-fold at the latest time point. Individuals experiencing more severe COVID-19 (severity 4–5) consistently showed higher neutralization titres against the VOCs compared with those experiencing milder disease (severity 0–3) (Fig. 2e).

Overall, wave 1 sera showed neutralizing activity against B.1.1.7, P.1 and B.1.351, albeit at a lower potency for B.1.1.7 and B.1.351.

### B.1.1.7 variant sera neutralizes other variants

**Fig. 3:**
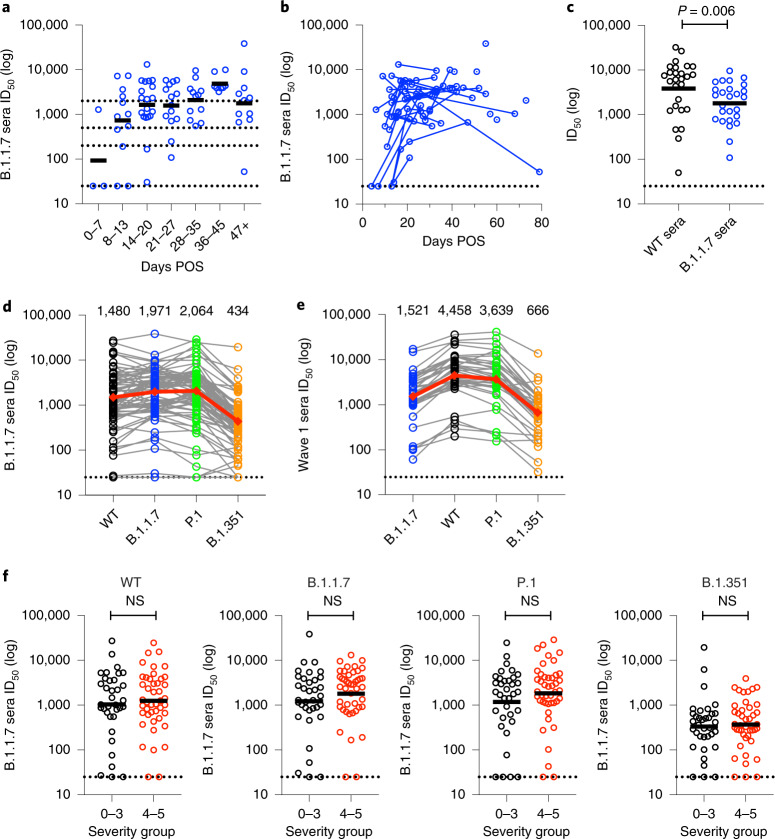
Neutralizing antibody responses in individuals infected with B.1.1.7. **a**, Serum neutralization against B.1.1.7 spike pseudotyped virus at different time windows (*n* = 79). The black lines represent the GMT. **b**, Neutralization of B.1.1.7 spike pseudotyped virus by sequential serum samples. Longitudinal samples from the same donor (*n* = 38 donors) are connected by a line. **c**, Comparison of homologous neutralization (that is, neutralization of WT pseudotyped virus by wave 1 sera (*n* = 27) and neutralization of B.1.1.7 pseudotyped virus by sera from B.1.1.7-infected individuals (*n* = 26)) at peak neutralization (21–35 days POS). Lines represent the GMT. *P* values were calculated using Mann–Whitney two-sided *U*-test. **d**, Cross-neutralizing activity of sera collected between days 10 and 60 POS from individuals infected with B.1.1.7 against four SARS-CoV-2 variants (*n* = 72). Each dot represents one individual, and matched data points for neutralization of different spike variants by the same serum sample are connected by lines. The red line represents the GMT against that virus (GMT values listed above graph columns). **e**, Cross-neutralizing activity of sera collected between days 10 and 60 POS from individuals infected in wave 1 against four SARS-CoV-2 variants (*n* = 35). Each set of dots connected by lines represents a serum sample. The red line represents the GMT against that virus (GMT values listed above graph columns). **f**, Comparison of the neutralization potency of B.1.1.7 sera against SARS-CoV-2 variants between individuals experiencing disease severity 0–3 (black, *n* = 35) or 4–5 (red, *n* = 44). The black lines represent the GMTs. *P* values were calculated using Mann–Whitney two-sided *U*-test. The horizontal dotted line represents the cut-off for the assay. Source data

**Extended Data Fig. 2:**
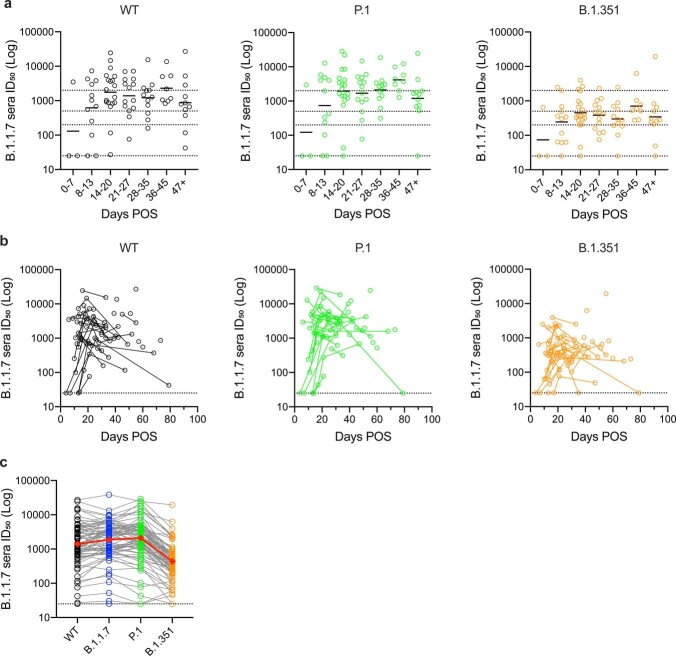
Cross-neutralizing antibody response in individuals infected with B.1.1.7. **a**) Serum neutralization against WT, P.1 and B.1.351 pseudotyped virus at different time windows (n = 79). Black line represents the geometric mean titre. **b**) Neutralization of WT, P.1 and B.1.351 pseudovirus by sequential serum samples. Longitudinal samples from the same donor (n = 38 donors) are connected by a line. **c**) Cross-neutralizing activity of all sera collected (days 4-79 POS) from individuals infected with B.1.1.7 against four SARS-CoV-2 variants (n = 79). Each line represents a serum sample. Red line represents the geometric mean titre against that virus. Source data

During the second wave of COVID-19 in December 2020 to February 2021 in the United Kingdom, the predominant variant infecting patients at St Thomas’ Hospital in London was B.1.1.7. Whole-genome sequencing was used to confirm infection with this lineage, and corresponding serum samples (*n* = 79) were collected from 38 individuals between 4 and 79 days POS at multiple time-points where possible. Homologous neutralization and cross-neutralizing activity were measured against WT, P.1 and B.1.351 pseudotyped particles (Fig. 3a and Extended Data Fig. 2a).

Sera from individuals infected with B.1.1.7 showed potent homologous neutralization (Fig. 3a). Analysis of both serially collected samples (Fig. 3b) and cross-sectional samples (Fig. 3a) showed that the neutralization of the B.1.1.7 variant followed similar kinetics, with the highest neutralization titres being detected around 3–5 weeks POS. For sera collected near the peak of the antibody response (21–35 days POS), more potent homologous neutralization was observed for wave 1 sera than B.1.1.7 sera (Fig. 3c); that is, a higher GMT was observed for wave 1 sera against WT pseudotyped particles compared with B.1.1.7 sera against B.1.1.7 pseudotyped particles. This may be indicative of either a higher immunogenicity of the WT spike protein compared with the B.1.1.7 spike protein, lower viral loads in B.1.1.7-infected individuals or of increased administration of immunosuppressive drugs, for example, dexamethasone during the second wave of COVID-19 in the United Kingdom^54^.

The majority of B.1.1.7 sera showed cross-neutralizing activity against the other VOCs (Extended Data Fig. 2a–c). Similar to wave 1 sera, the lowest cross-neutralization activity was observed against B.1.351, which exhibited an average 5.7-fold reduction in neutralizing activity compared with neutralization against B.1.1.7 across all the samples studied. Neutralization of P.1 and WT were reduced by an average 1.2-fold and 1.7-fold, respectively, compared with B.1.1.7. To enable a fair comparison of cross-neutralizing activity generated by infection with WT or B.1.1.7 virus, neutralization potency against the four viruses was compared for all sera collected between days 10 and 60 POS (Fig. 3d). Both B.1.1.7 sera (Fig. 3d) and wave 1 sera (Fig. 3e) showed a reduction in neutralization of B.1.351 compared with homologous neutralization of WT and B.1.1.7 pseudotypes (average 5.9-fold and 8.3-fold, respectively). Neutralization of P.1 by either wave 1 or B.1.1.7 sera was largely unchanged (1.3-fold and 1.2-fold changes, respectively). However, in contrast to convalescent sera from wave 1 that had an average 3.3-fold reduction in B.1.1.7 neutralization, there was only an average 1.7-fold reduction in WT neutralization by B.1.1.7 sera. This suggests that neutralization is retained against earlier lineage variants if infected with B.1.1.7.

**Extended Data Fig. 3:**
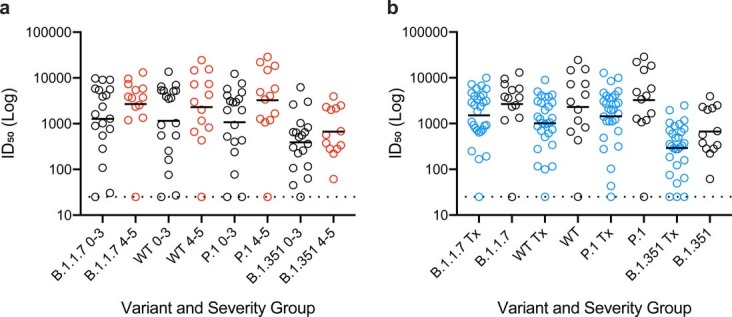
Neutralization titres in B.1.1.7 infected individuals with/without dexamethasone treatment. **a)** Comparison of the cross-neutralizing activity between 0-3 (black, n = 19) and 4-5 (red, n = 13) severity groups for B.1.1.7 infected individuals who had not yet received dexamethasone treatment at the time of serum sampling. Difference between 0-3 and 4-5 disease severity groups was calculated using a Mann–Whitney two-sided test *U*-test and showed no significant differences. **b)** Comparison of the cross-neutralizing activity for sera from B.1.1.7 infected individuals experiencing 4-5 disease, either having received (blue, n = 29) or not received (black, n = 13) dexamethasone treatment. Difference between treated and untreated groups were calculated using a Mann–Whitney two-sided test *U*-test and showed no significant differences. Source data

As we had previously observed a correlation between disease severity and neutralization titre for wave 1 sera (Fig. 2e), we similarly compared the GMTs for those with 0–3 or 4–5 disease severity for all B.1.1.7 serum samples. In contrast to wave 1 sera, the sera from B.1.1.7-infected individuals experiencing 4–5 disease severity did not display such an enhanced neutralization potency compared with the less severe group (severity 0–3) (Fig. 3f), which may also reflect the increased administration of immunosuppressive drugs during treatment. Indeed, when considering only those who had not received dexamethasone treatment before serum sampling^54^, a trend towards higher neutralization titres was observed for the 4–5 disease severity group compared with the 0–3 group (Extended Data Fig. 3a). Similarly, when focusing on the 4–5 disease severity group, higher GMTs were observed in those who had not received dexamethasone treatment (Extended Data Fig. 3b).

Overall, sera from individuals infected with the B.1.1.7 variant displayed potent cross-neutralizing activity.

### B.1.351 and B.1.617.2 spike proteins are antigenically distant

**Fig. 4:**
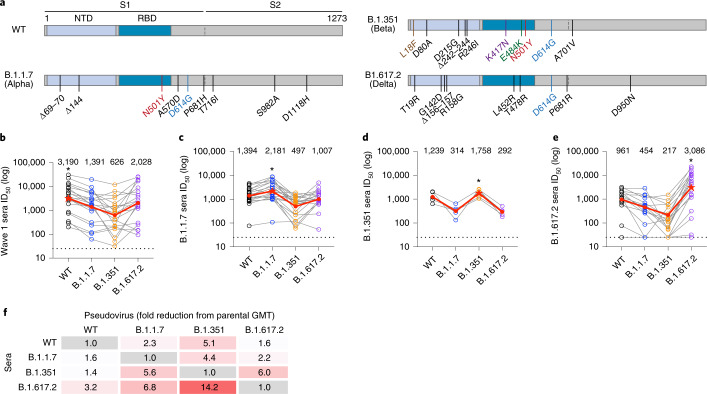
Cross-neutralizing activity in sera from individuals infected with WT, B.1.1.7, B.1.351 or B.1.617.2 SARS-CoV-2 variants. **a**, Schematic showing the position of spike mutations in the B.1.1.7, B.1.351 and B.1.617.2 variants. The major spike domains are indicated. **b**–**e**, Cross-neutralizing activity of sera from individuals infected in wave 1 (**b**; *n* = 20), with B.1.1.7 (**c**; *n* = 20), with B.1.351 (**d**; *n* = 5) or with B.1.617.2 (**e**; *n* = 20) against SARS-CoV-2 VOCs. Sera were collected in the acute phase of infection (wave 1 at 12–53 days POS, B.1.1.7 at 11–33 days POS, B.1.351 at 26–52 days POS, and B.1.617.2 at 12–32 days POS) and matched between groups. The dotted line represents the cut-off for the assay. The asterisk shows homologous neutralization. Each dot represents one individual, and matched data points for neutralization of different spike variants by the same serum sample are connected by lines. The red line represents the GMT against that virus, which is also reported as numerical values at the top of each graph. **f**, The fold reduction in GMT of sera from individuals infected with the different variants against WT, B.1.1.7, B.1.351 and B.1.617.2. Source data

Owing to the rapid spread of B.1.617.2 globally and the continued threat of B.1.351 emergence, cross-neutralization of these variants by immune sera is currently of particular importance, as well as the potential cross-protection provided following infection with B.1.617.2 or B.1.351. Therefore, to gain further insight into the antigenic distance between the spike glycoprotein of different SARS-CoV-2 VOCs, sera were collected from patients with COVID-19 at St Thomas’ hospital who had confirmed B.1.351 (*n* = 3) or B.1.617.2 (*n* = 20) infection, and cross-neutralizing activity was determined alongside a matched selection of wave 1 (*n* = 20) and B.1.1.7 (*n* = 20) sera. To enable a meaningful comparison, neutralization against WT, B.1.1.7, B.1.351 and B.1.617.2 variants (Fig. 4a) was measured for acute-phase serum samples collected 11–53 days POS (Fig. 4b–e). The convalescent sera from infection with each of the four viruses generated a cross-neutralizing antibody response, with the most potent neutralization observed against the homologous spike variant (Fig. 4b–e). The smallest reduction in potency compared with homologous neutralization was observed for virus particles pseudotyped with the parental WT spike protein across all convalescent serum groups. In contrast, a larger reduction in neutralization was observed against viral lineages that had evolved independently, which demonstrates that the B.1.1.7, B.1.351 and B.1.617.2 lineages are antigenically distinct (Fig. 4f). For wave 1, B.1.1.7 and B.1.617.2 sera, the greatest reduction in serum neutralization was against B.1.351. Infection with B.1.617.2 gave very potent homologous neutralization, but an average fold decrease in ID_50_ of 6.8 and 14.2 was observed against B.1.1.7 and B.1.351, respectively.

Overall, infection with newly emerged SARS-CoV-2 variants generates potent homologous neutralization, and neutralization of the parental WT is largely maintained across lineages. However, the spike proteins of the independent SARS-CoV-2 lineages are antigenically distant.

## Discussion

There is limited information on the longevity of the antibody response following natural infection with SARS-CoV-2 or COVID-19 vaccination. Initial concerns were that the SARS-CoV-2 antibody response might mimic that of other human endemic coronaviruses, such as 229E, for which antibody responses are short-lived and re-infections occur^55,56^. However, our data and that of other recent studies^35,57–63^ show that although neutralizing antibody titres decline from an initial peak response, robust neutralizing activity against both pseudotyped viral particles and infectious virus can still be detected in a large proportion of convalescent sera at up to 10 months POS. As IgM has been shown to facilitate neutralization^8,64^, the initial decline in neutralization is probably in part due to the reduction in circulating serum IgM observed, as well as the death of short-lived antibody-secreting cells, with the sustained neutralizing activity therefore arising from long-lived plasma cells producing spike-reactive IgG^3,58,65^. We observed a more notable decline in IgG responses to the nucleoprotein compared with IgG responses to the spike protein, which has also been observed by others^58^. This is particularly relevant when considering using IgG responses to the nucleoprotein to determine prior SARS-CoV-2 infection in COVID-19 vaccination studies. Further assessment of the longevity of the neutralizing antibody response arising from SARS-CoV-2 natural infection will become increasingly difficult as more of the global population receive a COVID-19 vaccine.

Although sustained neutralization against the infecting SARS-CoV-2 variant is important, efficacious cross-neutralizing activity is essential for long-term protection against emerging SARS-CoV-2 variants. In accordance with other recent reports, cross-neutralizing activity of wave 1 sera against viral variants was observed^34,38,39^. Despite a 3.4-fold and 8.9-fold reduction in neutralization potency against B.1.1.7 and B.1.351, respectively, high GMTs (3,331 and 1,303, respectively) were still observed at the neutralization peak, and neutralization of pseudotyped virus (that is, ID_50_ > 25) was detected in 17 out of 19 and in 18 out of 19 individuals at 257–305 days against B.1.1.7 and B.1.351, respectively. Interestingly, the differential neutralization of B.1.351 and B.1.1.7 compared with WT virus decreased at later time points for wave 1 sera, which suggests that antibodies present at later time points are better able to tolerate spike mutations. Indeed, a study by Gaebler et al.^22^ showed that SARS-CoV-2 mAbs isolated 6-months POS had more somatic hypermutation and displayed a greater resistance to RBD mutations. These observations suggest that COVID-19 vaccine boosting could be an important step for increasing both neutralization breadth and vaccine efficacy against newly emerging SARS-CoV-2 VOCs.

A current global concern is the efficacy of vaccines against B.1.617.2, which is driving the current wave of SARS-CoV-2 infections in the United Kingdom and globally. Acute-phase wave 1 sera showed cross-neutralization against B.1.617.2, with a 1.6-fold reduction in GMT compared with WT. Whether the reduced neutralizing antibody titres against viral variants reported here will be sufficient to protect against infection and/or severe disease is not fully understood^3–6,66^. Numerous studies have reported reduced neutralization of VOCs, in particular B.1.351, by sera from COVID-19 vaccinees^23,25,26,33,34,36–38,40^. Although a lower vaccine efficacy has been suggested in locations where B.1.351 is prevalent^67,68^, protection against B.1.1.7 infection has been reported in Israel following vaccination with BNT162b2 (ref. ^69^) and following AZD1222 in the United Kingdom^70^, and protection against symptomatic disease with B.1.617.2 following BNT162b2 vaccination in the United Kingdom^71^.

Spike proteins from VOCs are being investigated as second-generation vaccine candidates to tackle the challenges associated with protection against emerging variants of SARS-CoV-2 (refs. ^44–47^). Studying the immune response to spike variants in natural infection can provide initial insights into the antigenic distance between lineages and their ability to elicit broad protection against emerging viral variants. We showed that infection with B.1.1.7, B.1.351 or B.1.617.2 elicits a robust homologous neutralizing antibody response. However, a reduction in neutralization was observed against other SARS-CoV-2 variants. The smallest reduction was seen against WT virus, which indicates that neutralizing antibodies arising from infection with B.1.1.7, B.1.351 or B.1.617.2 are able to maintain efficacy against the previously dominant parental SARS-CoV-2 variant. Cele et al.^29^ also showed that B.1.351 infection generated better cross-neutralizing activity against earlier viral variants. These findings contrast with Faulkner et al.^48^, who observed a large decrease in cross-neutralization of WT virus in B.1.1.7-infected individuals. However, Faulkner et al.^48^ used sera collected at around 11 days POS and, as discussed above, cross-neutralizing activity probably develops over time. The reduced neutralization potency observed against independent SARS-CoV-2 lineages highlights the antigenic distance between the current VOCs. In agreement with Liu et al.^33^, the greatest antigenic distance appears to be between B.1.351 and B.1.617.2, which do not share common mutations. Importantly, we showed that sera from B.1.617.2 infection has the largest reduction in neutralization of B.1.1.7 and B.1.351 in the acute phase (average 6.8-fold and 14.2-fold reduction in GMT, respectively), which indicates that infection with B.1.617.2, or a vaccine based on B.1.617.2, will probably have lower efficacy against B.1.351 infection. Overall, these data suggest that immunization with the parental WT spike protein will probably give the broadest antibody response against the current VOCs and any newly emerging lineages in COVID-19-vaccine-naive populations.

The spike mutations responsible for differential serum neutralization of VOCs is not fully understood. As the RBD has been identified as a major target for neutralizing antibodies, the RBD mutations K417T/N, E484K and N501Y are of particular concern for immune evasion, and these mutations lead to neutralization resistance for several RBD-specific mAbs under clinical development^22,28,72–74^. Additionally, mutations in the NTD also lead to neutralization resistance for some NTD-specific mAbs^20,28,75^. In contrast, neutralization by some RBD-specific mAbs and NTD-specific mAbs is unaffected by variation in the spike protein, thereby highlighting the presence of cross-neutralizing epitopes on both the RBD and the NTD^20,27,30–33,40^. In the present study, the most neutralization-resistant VOC was B.1.351. Wave 1 and B.1.1.7 sera showed an average 4.8-fold and 5.7-fold, respectively, ID_50_ reduction against B.1.351, which encodes the RBD mutations K417N, E484K and N501Y. Despite P.1 encoding similar RBD mutations (K417T, E484K and N501Y), only a minor decrease in neutralization potency was observed. Therefore, as these two VOCs also encode a different pattern of mutations in the NTD and the S2 domain of the spike protein, these combined data indicate that mutations in the RBD, the NTD and the S2 domain all contribute to the reduced serum neutralization potency and suggests that assessment of mutational profiles throughout all spike domains will be important when considering immune evasion by emerging viral variants^27^. In-depth analysis of the antibody response at the monoclonal level is required to understand this further.

In summary, using convalescent sera from individuals infected in wave 1, we showed that cross-neutralizing antibodies are detected up to 10 months POS in some individuals. Infection with B.1.1.7, B.1.351 or B.1.617.2 generates a cross-neutralizing antibody response that is effective against the parental virus but has reduced neutralization against divergent lineages. These findings highlight the antigenic distance between spike proteins of current VOCs and have implications for the optimization of COVID-19 vaccines that are effective at eliciting a cross-neutralizing antibody response that protects against the current and newly emerging SARS-CoV-2 variants.

## Methods

### Ethics

This research complies with all relevant ethical regulations. The ethical oversight for this continuing study was the same as for the original study^8^. Collection of surplus/discarded serum samples was approved by South Central REC 20/SC/0310. For sera collected from healthcare workers, signed, informed consent was obtained with expedited approval from the Guy’s and St Thomas’ NHS Foundation Trust R&D office, the occupational health department and the medical director.

### Patient samples

Some sera were previously studied in Seow et al.^8^ as stated in the manuscript. Additional discarded serum samples collected as part of routine hospital care were identified at time points >100 days POS from any individuals who were returning to hospital as part of their routine clinical care (a subset comprising 29 out of 59 participants), in addition to from healthcare workers still employed at St Thomas’ Hospital (a subset comprising 9 out of 37 participants). Overall, 64 serum samples were collected from 38 individuals (65.8% male and aged 23–83 years, median 50 years), including 16 serum samples collected between 145 and 175 days POS (TP3), 29 collected between 180 and 217 days (TP4) and 19 collected between 257 and 305 days POS (TP5).

SARS-CoV-2 cases were diagnosed by RT–PCR of respiratory samples at St Thomas’ Hospital, London. A total of 894 serum samples from 585 individuals were saved between 4 January 2020 and 12 March 2021 and between 22 June 2021 and 12 July 2021. Samples obtained ranged from 8 days before up to 79 days POS. Cases were linked to corresponding genome sequencing of viral isolates from nose and throat swabs. A total of 79 serum samples were collected from 38 individuals with a confirmed B.1.1.7 infection (52.6% male, aged 37–96 years, median 63 years). A total of 5 serum samples were collected from 3 individuals with a confirmed B.1.351 infection (100% male, aged 26–80 years). In addition, 20 serum samples were collected from 20 individuals with a confirmed B.1.617.2 infection (85% male, aged 23–82 years, median 36 years).

### Plasmids

The WT^8^ and B.1.1.7 (refs. ^20,24^) spike plasmids have been previously described. B.1.1.7 mutations introduced were ΔH69/V70, ΔY144, N501Y, A570D, D614G, P681H, T716I, S982A and D1118H. Spike genes encoding the variants B.1.351 and P.1 were synthesized (Genewiz) and cloned into pcDNA3.1. B.1.351 mutations introduced were L18F, D80A, D215G, Δ242–244, R246I, K417N, E484K, N501Y, D614G and A701V. P.1 mutations introduced were L18F, T20N, P26S, D138Y, R190S, K417T, E484K, N501Y, D614G, H655Y, T1027I and V1176F. B.1.617.2 spike plasmid was kindly provided by W. Barclay (Imperial College London) and mutations introduced were T19R, G142D, Δ156–157, R158G, L452R, T478R, D614G, P681R and D950N.

### COVID-19 severity classification

The score, ranging from 0 to 5, was devised to mitigate underestimating disease severity in patients not for escalation above level one (ward-based) care. Patients diagnosed with COVID-19 were classified as follows: 0, asymptomatic or no requirement for supplemental oxygen; 1, requirement for supplemental oxygen (fraction of inspired oxygen (*F*_i_O_2_) < 0.4) for at least 12 h; 2, requirement for supplemental oxygen (*F*_i_O_2_ ≥ 0.4) for at least 12 h; 3, requirement for noninvasive ventilation/continuous positive airway not a candidate for escalation above level one (ward-based) care; 4, requirement for intubation and mechanical ventilation or supplemental oxygen (*F*_i_O_2_ > 0.8) and peripheral oxygen saturations <90% (with no history of type 2 respiratory failure) or <85% (with known type 2 respiratory failure) for at least 12 h; and 5, requirement for extracorporeal membrane oxygenation.

### Viral sequencing

Whole-genome sequencing of residual nose-and-throat swabs from SARS-CoV-2 cases was performed using GridION (Oxford Nanopore Technology) and v.3 of the ARTIC protocol and bioinformatics pipeline^76^. From November 2020, all samples from in-patients were assessed for sequencing. Samples were selected for sequencing if the corrected CT value was 32 or below or the Hologic Aptima assay was above 1,000 RLU, and if there was sufficient residual sample. Sequencing was performed under COG-UK ethical approval. Lineage determination was performed using updated versions of pangolin 2.0 (ref. ^77^). Samples were regarded as successfully sequenced if over 50% of the genome was recovered and if lineage assignment by pangolin was given with at least 50% confidence.

### Glycoprotein expression and purification

The recombinant spike (Wuhan-1 strain) consists of a pre-fusion spike ectodomain at residues 1–1138 with proline substitutions at amino-acid positions 986 and 987, a GGGG substitution at the furin cleavage site (amino acids 682–685) and an N terminal T4 trimerization domain followed by a Strep-tag II (ref. ^21^). Spike protein was expressed in HEK-293 Freestyle cells and purified using StrepTactinXT Superflow high capacity 50% suspension according to the manufacturer’s protocol by gravity flow (IBA Life Sciences).

The RBD (residues 319–541) was joined to a carboxy-terminal hexahistidine tag. The protein was expressed HEK-293 Freestyle cells and purified using Ni-NTA agarose beads.

The nucleoprotein was obtained from the James Lab at LMB, Cambridge. The nucleoprotein is a truncated construct of the SARS-CoV-2 nucleoprotein comprising residues 48–365 with an N-terminal uncleavable hexahistidine tag. Nucleoprotein was expressed in *Escherichia* *coli* using autoinducing medium for 7 h at 37 °C and purified using immobilized metal affinity chromatography, size exclusion and heparin chromatography.

### ELISA binding to the nucleoprotein, the spike protein and the RBD

ELISAs were carried out as previously described^8,50^. All sera were heat-inactivated at 56 °C for 30 min before use. High-binding ELISA plates (Corning, 3690) were coated with antigen (nucleoprotein, spike glycoprotein or RBD) at 3 μg ml^–1^ (25 μl per well) in PBS either overnight at 4 °C or for 2 h at 37 °C. Wells were washed with PBS-T (PBS with 0.05% Tween-20) and then blocked with 100 μl of 5% milk in PBS-T for 1 h at room temperature. The wells were emptied, and serum diluted at 1:50 in milk was added and incubated for 2 h at room temperature. Wells were washed with PBS-T. Secondary antibody was added and incubated for 1 h at room temperature. IgM was detected using goat-anti-human-IgM-HRP (horseradish peroxidase) (1:1,000) (Sigma, catalogue no. A6907) and IgG was detected using goat-anti-human-Fc-AP (alkaline phosphatase) (1:1,000) (Jackson, catalogue no. 109-055-098). Wells were washed with PBS-T and either AP substrate (Sigma) was added and read at 405 nm (AP) or one-step 3,3′,5,5′-tetramethylbenzidine (TMB) substrate (Thermo Fisher Scientific) was added and quenched with 0.5 M H_2_S0_4_ before reading at 450 nm (HRP). Control reagents included CR3009 (2 μg ml^–1^), CR3022 (0.2 μg ml^–1^), negative control plasma (1:25 dilution), positive control plasma (1:50) and blank wells. ELISA measurements were performed in duplicate, and the mean of the two values was used.

### SARS-CoV-2 pseudotyped virus particle preparation

Pseudotyped HIV-1 virus incorporating the SARS-CoV-2 spike protein (WT, B.1.1.7, P.1, B.1.351 or B.1.617.2) was produced in a 10-cm dish seeded the day before with 5 × 10^6^ HEK293T/17 cells in 10 ml of complete Dulbecco’s modified Eagle’s medium (DMEM-C, 10% fetal bovine serum (FBS) and 1% penicillin–streptomycin) containing 10% (v/v) FBS, 100 IU ml^–1^ penicillin and 100 μg ml^–1^ streptomycin. Cells were transfected using 90 μg of PEI-Max (1 mg ml^–1^, Polysciences) with 15 μg of HIV-luciferase plasmid, 10 μg of HIV 8.91 gag/pol plasmid and 5 μg of SARS-CoV-2 spike protein plasmid^78,79^. The supernatant was collected 72 h after transfection. Pseudotyped virus particles were filtered through a 0.45-μm filter, purified by sucrose cushion ultracentrifugation and stored at –80 °C until required.

### Neutralization assay with SARS-CoV-2 pseudotyped virus

Serial dilutions of serum samples (heat-inactivated at 56 °C for 30 mins) were prepared with DMEM (25 µl) (10% FBS and 1% penicillin–streptomycin) and incubated with pseudotyped virus (25 µl) for 1 h at 37 °C in half-area 96-well plates. Next, HeLa cells stably expressing the ACE2 receptor were added (10,000 cells per 25 µl per well) and the plates were left for 72 h. Infection levels were assessed in lysed cells with a Bright-Glo luciferase kit (Promega) using a Victor X3 multilabel reader (Perkin Elmer). Each serum sample was run in duplicate and was measured against the four SARS-CoV-2 variants within the same experiment using the same dilution series.

### Infectious virus strain and propagation

Vero-E6 TMPRSS2 cells^53^ (*Cercopithecus aethiops*-derived epithelial kidney cells) were grown in DMEM (Gibco) supplemented with GlutaMAX, 10% FBS and 20 µg ml^–1^ gentamicin, and incubated at 37 °C with 5% CO_2_. SARS-CoV-2 strain England 2 (England 02/2020/407073) was obtained from Public Health England. The virus was propagated by infecting 60–70% confluent Vero-E6 TMPRSS2 cells in T75 flasks at a multiplicity of infection of 0.005 in 3 ml of DMEM supplemented with GlutaMAX and 10% FBS. Cells were incubated for 1 h at 37 °C before adding 15 ml of the same medium. Supernatant was collected 72 h after infection following visible cytopathic effect, and filtered through a 0.22-µm filter to eliminate debris, aliquoted and stored at −80 °C. The infectious virus titre was determined by plaque assay using Vero-E6 TMPRSS2 cells.

### Infectious virus neutralization assay

Vero-E6 TMPRSS2 cells^76^ were seeded at a concentration of 20,000 cells per 100 µl per well in 96-well plates in DMEM (10% FBS and 1% penicillin–streptomycin) and allowed to adhere overnight. Serial dilutions of mAbs were prepared with DMEM (2% FBS and 1% penicillin–streptomycin) and incubated with replication-competent live SARS-CoV-2 for 1 h at 37 °C. The medium was removed from the pre-plated Vero-E6 TMPRSS2 cells, and the serum–virus mixtures were added to the cells and incubated at 37 °C for 24 h. The virus–serum mixture was aspirated, and each well was fixed with 150 µl of 4% formalin at 4 °C overnight and then topped up to 300 µl using PBS. The cells were washed once with PBS and permeabilized with 0.1% Triton-X in PBS at room temperature for 15 min. The cells were washed twice with PBS and blocked using 3% milk in PBS at room temperature for 15 min. The blocking solution was removed and a nucleoprotein-specific mAb (murinized-CR3009)^80^ was added at 2 µg ml^–1^ (diluted using 1% milk in PBS) at room temperature for 45 min. The cells were washed twice with PBS and goat-anti-mouse-IgG-conjugated to HRP was added (1:3,000 in 1% milk in PBS, A2554-1 ml, Sigma-Aldrich) at room temperature for 1 h. The cells were washed twice with PBS, developed using TMB substrate for 30 min and quenched using 2 M H_2_SO_4_ before reading at 450 nm. Measurements were performed in duplicate and the duplicates were used to calculate the ID_50_.

### Statistical analysis

Analyses were performed using GraphPad Prism v.8.3.1.

### Reporting Summary

Further information on research design is available in the Nature Research Reporting Summary linked to this article.

## Supplementary information

Reporting Summary

## Data Availability

The authors declare that the data supporting the findings of this study are available within the paper and its supplementary information files. Source data are provided with this paper.

## Source data

Source Data Fig. 1 Data points from Prism graphs.
Source Data Fig. 2 Data points from Prism graphs.
Source Data Fig. 3 Data points from Prism graphs.
Source Data Fig. 4 Data points from Prism graphs.
Source Data Extended Data Fig. 1 Data points from Prism graphs.
Source Data Extended Data Fig. 2 Data points from Prism graphs.
Source Data Extended Data Fig. 3 Data points from Prism graphs.
