# Supplementary material for: Neutralizing antibody activity in convalescent sera from infection in humans with SARS-CoV-2 and variants of concern
Source: Nat Microbiol. 2021 Oct 15;6(11):1433–42. doi: 10.1038/s41564-021-00974-0 (PMC8556155; doi:10.1038/s41564-021-00974-0)
Supplement: Supplementary file 1 — Reporting Summary [file 41564_2021_974_MOESM1_ESM.pdf]

nature portfolio

Corresponding author(s): Katie Doores

Last updated by author(s): Aug 26, 2021

Reporting Summary

Nature Portfolio wishes to improve the reproducibility of the work that we publish. This form provides structure for consistency and transparency in reporting. For further information on Nature Portfolio policies, see our Editorial Policies and the Editorial Policy Checklist.

Statistics

For all statistical analyses, confirm that the following items are present in the figure legend, table legend, main text, or Methods section.

n/a	Confirmed	
☐	☒	The exact sample size (*n*) for each experimental group/condition, given as a discrete number and unit of measurement
☐	☒	A statement on whether measurements were taken from distinct samples or whether the same sample was measured repeatedly
☐	☒	The statistical test(s) used AND whether they are one- or two-sided<br>*Only common tests should be described solely by name; describe more complex techniques in the Methods section.*
☒	☐	A description of all covariates tested
☒	☐	A description of any assumptions or corrections, such as tests of normality and adjustment for multiple comparisons
☐	☒	A full description of the statistical parameters including central tendency (e.g. means) or other basic estimates (e.g. regression coefficient) AND variation (e.g. standard deviation) or associated estimates of uncertainty (e.g. confidence intervals)
☐	☒	For null hypothesis testing, the test statistic (e.g. *F*, *t*, *r*) with confidence intervals, effect sizes, degrees of freedom and *P* value noted<br>*Give P values as exact values whenever suitable.*
☒	☐	For Bayesian analysis, information on the choice of priors and Markov chain Monte Carlo settings
☒	☐	For hierarchical and complex designs, identification of the appropriate level for tests and full reporting of outcomes
☐	☒	Estimates of effect sizes (e.g. Cohen's *d*, Pearson's *r*), indicating how they were calculated

Our web collection on statistics for biologists contains articles on many of the points above.

Software and code

Policy information about availability of computer code

Data collection	No software was used for data collection.
Data analysis	GraphPad Prism v8 and Pangolin v2 were used for data analysis.

For manuscripts utilizing custom algorithms or software that are central to the research but not yet described in published literature, software must be made available to editors and reviewers. We strongly encourage code deposition in a community repository (e.g. GitHub). See the Nature Portfolio guidelines for submitting code & software for further information.

Data

Policy information about availability of data

All manuscripts must include a data availability statement. This statement should provide the following information, where applicable:

- Accession codes, unique identifiers, or web links for publicly available datasets
- A description of any restrictions on data availability
- For clinical datasets or third party data, please ensure that the statement adheres to our policy

The authors declare that the data supporting the findings of this study are available within the paper and source data files.

Field-specific reporting

Please select the one below that is the best fit for your research. If you are not sure, read the appropriate sections before making your selection.

☒ Life sciences ☐ Behavioural & social sciences ☐ Ecological, evolutionary & environmental sciences

For a reference copy of the document with all sections, see nature.com/documents/nr-reporting-summary-flat.pdf

Life sciences study design

All studies must disclose on these points even when the disclosure is negative.

Sample size	Sample size for wave 1 was chosen based on availability of serum samples collected at multiple time points from patients admitted to St Thomas' Hospital, London. This included longitudinal samples from 29 patients and 9 healthcare workers at time points 1-305 days post onset of symptoms. For this study, an additional 64 sera were collected from 38 individuals >100 days POS.<br>79 sera were collected from 38 individuals with a confirmed B.1.1.7 infection.<br>5 sera were collected from 3 individuals with a confirmed B.1.351 infection.<br>20 sera were collected from 20 individuals with a confirmed B.1.617.2 infection.<br>The size of these groups were determined by sera availability.
Data exclusions	All data was included in the analysis.
Replication	The reproducibility of the ELISA assays was confirmed in a previous study. Reproducibility of neutralization ID50 were measured for a subset of serum samples (n = 20, with similar results) but due to low volumes of serum samples available we could not perform this for all samples.
Randomization	Randomization was not relevant to this study as this was an observational study.
Blinding	Blinding was not relevant to this study because of the observational design. Blinded samples were used in assay development.

Reporting for specific materials, systems and methods

We require information from authors about some types of materials, experimental systems and methods used in many studies. Here, indicate whether each material, system or method listed is relevant to your study. If you are not sure if a list item applies to your research, read the appropriate section before selecting a response.

Materials & experimental systems

n/a	Involved in the study	
☐	☒	Antibodies
☐	☒	Eukaryotic cell lines
☒	☐	Palaeontology and archaeology
☒	☐	Animals and other organisms
☐	☒	Human research participants
☒	☐	Clinical data
☒	☐	Dual use research of concern

Methods

n/a	Involved in the study	
☒	☐	ChIP-seq
☒	☐	Flow cytometry
☒	☐	MRI-based neuroimaging

Antibodies

Antibodies used	Secondary antibodies:<br>Goat-anti-Human-IgM HRP (Sigma, A6907, Lot number: 147522)<br>Goat-anti-human-Fc-AP (Jackson: 109-055-098-JIR, Lot number: SLCC6480)<br>Goat-anti-mouse-Fc-HRP (Sigma, A2554-1mL, Lot number: 045M4780V)<br><br>Primary antibodies expressed in-house:<br>CR3022 (Spike and RBD specific mAb)<br>CR3009 (N specific mAb)
Validation	All secondary antibodies used are commercial antibodies reported by the manufacturer to be validated for use in ELISA.<br>CR3022 and CR3009 positive control antibodies were validated in Pickering et al PLoS Pathog. 2020 Sep 24;16(9):e1008817.

Eukaryotic cell lines

Policy information about cell lines

Cell line source(s)	HEK 293T-17 cells (ATCC)

Cell line source(s)	HEK 293F Cells (Thermofisher)<br>HeLa ACE2 cells (Produced by Dr James Voss, Scripps)<br>Vero-E6 TMPRSS2 cells (produced by Prof Stuart Neil, KCL).
Authentication	No authentication was performed. All expression constructs were Sanger sequenced.
Mycoplasma contamination	These cell lines tested negative for mycoplasma.
Commonly misidentified lines<br>(See ICLAC register)	No commonly misidentified cell lines were used.

Human research participants

Policy information about studies involving human research participants

Population characteristics	The cohort consists of patients previously admitted to St Thomas' Hospital for treatment of COVID-19 (n = 90) and Health care workers (n = 9). Overall, 65.7% were male and 34.3% were female and ages ranged from 23-96 years (median 56 years).
Recruitment	No participants were enrolled. All samples pre-existed.
Ethics oversight	Collection of surplus samples was approved by the South Central REC 20/SC/0310. SARS-CoV-2 cases were diagnosed by RT-PCR of respiratory samples at St Thomas' Hospital, London.

Note that full information on the approval of the study protocol must also be provided in the manuscript.
